# Characterization of cysteine proteases from poultry red mite, tropical fowl mite, and northern fowl mite to assess the feasibility of developing a broadly efficacious vaccine against multiple mite species

**DOI:** 10.1371/journal.pone.0288565

**Published:** 2023-07-13

**Authors:** Shwe Yee Win, Shiro Murata, Sotaro Fujisawa, Hikari Seo, Jumpei Sato, Yoshinosuke Motai, Takumi Sato, Eiji Oishi, Akira Taneno, Lat Lat Htun, Saw Bawm, Tomohiro Okagawa, Naoya Maekawa, Satoru Konnai, Kazuhiko Ohashi

**Affiliations:** 1 Laboratory of Infectious Diseases, Department of Disease Control, Faculty of Veterinary Medicine, Hokkaido University, Kita-ku, Sapporo, Japan; 2 Department of Advanced Pharmaceutics, Faculty of Veterinary Medicine, Hokkaido University, Kita-ku, Sapporo, Japan; 3 Vaxxinova Japan K.K., Minato-ku, Tokyo, Japan; 4 Department of Pharmacology and Parasitology, University of Veterinary Science, Yezin, Nay Pyi Taw, Myanmar; 5 Department of Livestock and Aquaculture Research, Ministry of Agriculture, Livestock and Irrigation, Nay Pyi Taw, Myanmar; 6 International Affairs Office, Faculty of Veterinary Medicine, Hokkaido University, Kita-ku, Sapporo, Japan; Beni Suef University Faculty of Veterinary Medicine, EGYPT

## Abstract

Infestation with poultry red mites (PRM, *Dermanyssus gallinae*) causes anemia, reduced egg production, and death in serious cases, resulting in significant economic losses to the poultry industry. As a novel strategy for controlling PRMs, vaccine approaches have been focused upon and several candidate vaccine antigens against PRMs have been reported. Tropical (TFM, *Ornithonyssus bursa*) and northern (NFM, *Ornithonyssus sylviarum*) fowl mites are also hematophagous and cause poultry industry problems similar to those caused by PRM. Therefore, ideal antigens for anti-PRM vaccines are molecules that cross-react with TFMs and NFMs, producing pesticidal effects similar to those against PRMs. In this study, to investigate the potential feasibility of developing vaccines with broad efficacy across mite species, we identified and characterized cysteine proteases (CPs) of TFMs and NFMs, which were previously reported to be effective vaccine antigens of PRMs. The open reading frames of CPs from TFMs and NFMs had the same sequences, which was 73.0% similar to that of PRMs. Phylogenetic analysis revealed that the CPs of TFMs and NFMs clustered in the same clade as CPs of PRMs. To assess protein functionality, we generated recombinant peptidase domains of CPs (rCP-PDs), revealing all rCP-PDs showed CP-like activities. Importantly, the plasma obtained from chickens immunized with each rCP-PD cross-reacted with rCP-PDs of different mites. Finally, all immune plasma of rCP-PDs reduced the survival rate of PRMs, even when the plasma was collected from chickens immunized with rCP-PDs derived from TFM and NFM. Therefore, CP antigen is a promising, broadly efficacious vaccine candidate against different avian mites.

## 1 Introduction

Poultry red mites (PRMs), *Dermanyssus gallinae*, northern fowl mites (NFM), *Ornithonyssus sylviarum*, and tropical fowl mites (TFMs), *Ornithonyssus bursa*, are obligatory hematophagous ectoparasites of poultry, including chickens. The mites are endemic across the Asian continent including Japan, Myanmar, China, and Vietnam. PRMs and NFMs are largely distributed in America and Europe [1–4]. PRM takes a blood meal and lives off-host in cracks and crevices of the poultry house. In contrast, the entire life cycle of TFMs and NFMs occurs on the host [3, 5]. Infestation of chickens with these mites causes stress-induced immunosuppression, blood loss, anemia, and death in serious cases. These effects result in substantial economic losses to the poultry industry [4, 6, 7] and negatively impact animal welfare [5]. Furthermore, the mites are of veterinary and medical importance due to their potential role in the transmission of avian pathogens [8] and tendency to bite humans, which causes itching and dermatitis [9].

Although the control of avian mites mainly relies on the use of acaricides, prolonged use of acaricides and suboptimal level of acaricides have led to the emergence of acaricide-resistant mites, resulting in reduced efficacy of the acaricides in controlling these mites [10, 11]. As for PRMs, the ability of the mites to hide in cracks and crevices after feeding on blood protects them against acaricide exposure [7]. In addition, there are concerns regarding food and environmental contamination associated with the use of acaricides. To overcome these problems, alternative control methods for avian mites are needed. The following alternative methods have been considered for controlling PRMs: vaccine approaches [12–20], the application of essential oils and plant extracts, and the introduction of biological predators [21, 22]. Among these, we focused on vaccine approaches as a protective strategy against PRMs [12, 15–18]. Though previous studies have shown the potential usefulness of vaccines, their efficacy needs to be improved for practical applications [23]. Vaccines are acceptable to the poultry industry and offer the following advantages over existing treatments: not toxic to the environment, efficacy is unlikely to be affected by resistance, cost-effective, and long lasting protection [24]. PRMs, TFMs and NFMs have similar morphological and biological characteristics. Therefore, the identification of highly conserved molecules among these mite species may facilitate the development of a universal vaccine with broad-spectrum efficacy against avian mites.

The development of anti-PRM vaccines relies on the identification of molecules with crucial physiological functions in PRMs that can act as protective antigens to which the host develops an immune response. In addition, preferred antigens for universal vaccines are molecules associated with properties common to avian mites. Many molecules tested as vaccine molecules in PRMs include enzymes such as cysteine proteases (CPs) 1 and 2 [12, 14, 18] and cathepsin D- and L-like proteases [13], possibly related to hemoglobin digestion. Use of these vaccine antigens may affect the mortality of PRMs [13, 14, 18, 20]. Our and another group have previously reported the potential usefulness of *Dermanyssus gallinae* cysteine protease 1 (Deg-CPR-1) as a vaccine antigen [14, 18]. Cysteine proteases (CPs) are proteolytic enzymes involved in biological processes such as catabolism and protein processing. They are present in various organisms from viruses, bacteria, and parasites to higher organisms such as mammals. In humans, there are 11 CPs, including cathepsins B, C, F, H, K, L, O, S, V, X, and W, as shown via bioinformatics analysis of the human genome [25]. CPs have been characterized in some parasites. Further, their involvement in hemoglobin degradation, parasite egress, and surface protein processing in *Plasmodium falciparum* has been shown [26]. In addition, cathepsins B, C, and L are reportedly involved in hemoglobin digestion in some ticks [27]; therefore, they are considered potentially effective antigens for anti-tick vaccines [28]. Blood feeding is a common characteristic of PRMs, TFMs, and NFMs, and is essential for mite growth. Proteases with similar characteristics are potentially conserved among these avian mite species; therefore, they are potentially useful as antigens for the development of universal vaccines against PRMs, TFMs, and NFMs. In this study, we evaluated the potential usefulness of CPs as antigens for universal vaccines against avian mites.

## 2 Materials and methods

### 2.1 Ethics statement

All animal experiments were approved by the Institutional Animal Care and Use Committee of Hokkaido University (approval number:20–0051) and followed the relevant guidelines and regulations of the Faculty of Veterinary Medicine, Hokkaido University, which is fully accredited by the Association for Assessment and Accreditation of Laboratory Animal Care International (AAALAC).

### 2.2 Sample availability

For this study, samples that were morphologically and genetically characterized as TFMs and NFMs collected in Myanmar were used [29]. PRMs were collected from an egg-laying farm in Japan using a TubeSpin Bioreactor 600 bottle (TPP Techno Plastic Products AG, Trasadingen, Switzerland) and transferred to the laboratory at 4°C. PRMs were stored at 5°C until use. Thereafter, they were kept at 25°C for a week without blood feeding.

### 2.3 RNA extraction and complementary DNA (cDNA) synthesis

Total RNA was extracted from each mite species using TRIzol reagent (Invitrogen, Carlsbad, CA, USA) according to the manufacturer’s instructions. Samples were treated with DNase I (Invitrogen, Carlsbad, CA) to remove unwanted DNA. Then, cDNA was synthesized from 1 μg of total RNA using PrimeScript Reverse Transcriptase (Takara Bio Inc., Shiga, Japan) and 200 pmol of oligo (dT)18 primer (Hokkaido System Science, Hokkaido, Japan).

### 2.4 Rapid amplification of cDNA ends (RACE) and molecular cloning of CP genes

To characterize *CP* genes of TFMs and NFMs, we amplified a segment of *CPs* via nested PCR using primers shown in S1 Table. Primers were designed based on nucleotide sequences of a conserved region of *CPs* of *Dermanyssus gallinae* (HZ459284, KR697573) and other mite species including *Varroa destructor* (XM 022808259, XM 022835169), and *Metaseiulus occidentalis* (XM 018640260). Amplified fragments were cloned into a pGEMT easy vector (Promega, Madison, WI, USA) and nucleotide sequences were analyzed using a Beckman CEQ GeXP automated sequencer (Beckman Coulter Inc., Brea, CA). Based on partial sequences of *CPs* of TFMs and NFMs, we designed primers for 3′ and 5′ RACE to amplify the open reading frames (ORFs) of *CPs*. We performed 3′ and 5′ RACE PCR using the RACE system (Invitrogen) in accordance with the manufacturer’s protocol. 5′ and 3′ RACE PCR products were separated via agarose gel electrophoresis, purified, cloned into the pGEMT-Easy vector (Promega), and transformed into competent DH5α *Escherichia coli* cells.

### 2.5 Genetic analysis

ORFs of *CPs* from PRMs, TFMs, and NFMs were genetically characterized using the Basic Local Alignment Search Tool (BLAST) to assess homology with other species (S2 Table). For phylogenetic analysis, nucleotide sequences of *CP* genes of arthropods including other mites and ticks, chickens, and other species were aligned using the MUSCLE (codon) option of MEGA X software [30]. A maximum-likelihood phylogenetic tree was constructed using the same software with 1,000 bootstrap replicates and a discrete gamma distribution (+G) to improve tree topology.

### 2.6 Expression of recombinant cysteine protease-peptidase domain (rCP-PD) proteins

PRM, TFM, and NFM N-terminal His-tagged rCP-PD proteins were generated using *E*. *coli* expression systems designated as rCP-PD PRM, rCP-PD TFM, and rCP-PD NFM, respectively. For rCP-PD PRM, the reference sequence of *CP* (HZ459284) from Japan was used to express the PD of the PRM CP protein. Coding regions of CP-PDs were amplified using primers containing NdeI and XhoI restriction sites (S1 Table). The amplified products were cloned into a pET19b vector (Merck & Co., Inc., Rahway, NJ, USA) and transformed into *E*. *coli* (DE3, pLysS) (Merck). Recombinant protein expression and purification was performed according to the manufacturer’s instructions. Bacterial pellets were separated into soluble and insoluble fractions using BugBuster solution (Merck), with insoluble fractions solubilized in buffer containing 0.3% N-lauroylsarcosine and 50 mM N-cyclohexyl-3-aminopropanesulfonic acid (CAPS; Merck) (pH 11.0). Recombinant proteins were purified using Ni Sepharose™ 6 Fast Flow resin (GE Healthcare, Chicago, IL, USA) and eluted with 0.3% N-lauroylsarcosine, 50 mM CAPS (pH 11.0) and 250 mM imidazole (Nacalai Tesque, Tokyo, Japan). Eluted recombinant proteins were dialyzed with 10 mM Tris-HCL (pH 8.5) buffer containing 0.1 mM DL-Dithiothreitol (DTT) (Merck) and allowed to refold at 4°C overnight. Recombinant proteins were concentrated using a 10 K centrifugal filter unit (Merck). Recombinant protein concentration was determined using a Pierce™ Bicinchoninic Acid Protein Assay Kit (Thermo Fisher Scientific, Waltham, MA, USA) according to the manufacturer’s instructions. To confirm recombinant protein expression and purification, they were denatured in 2× sodium dodecyl sulfate (SDS) buffer (125 mM Tris-HCl [pH 6.8], 4% SDS, 10% 2-mercaptoethanol, and 20% glycerol), heat-treated at 96°C for 5 min, separated via 13% SDS-polyacrylamide gel electrophoresis (SDS-PAGE), and stained with Coomassie Brilliant Blue (FUJIFILM Wako Pure Chemical Corporation, Osaka, Japan).

### 2.7 Immunization of chickens with rCP-PD proteins

To generate immune plasma, chickens were immunized with each of the rCP-PDs. rCP-PDs were mixed with Freund’s incomplete adjuvant (FUJIFILM Wako Pure Chemical Corporation). Four chickens per group were subcutaneously immunized with 20 μg of each rCP-PD generated, at 3 weeks of age. Three weeks after the first immunization, recombinant proteins with the same adjuvant were used to perform a second immunization. As a control, four chickens were immunized with PBS mixed with the same adjuvant. Chickens were euthanized by collecting heparinized whole blood from the hearts under deep general anesthesia by inhaling Isoflurane (Zoetis Japan, Tokyo, Japan) 3 weeks after the 2^nd^ immunization, and immune plasma was isolated from the whole blood samples. Throughout the experimental period, we monitored the health status of chickens and observed no deaths, weight loss, or other clinical signs in all immunized groups.

### 2.8 Western blotting

Western blotting was performed to confirm the production of specific antibodies against rCP-PD PRM, rCP-PD TFM, and rCP-PD NFM, and to analyze the cross-reactivity of immunized plasma. rCP-PD proteins were separated via 13% SDS-PAGE and transferred to polyvinylidene difluoride membranes (Merck). Membranes were blocked with 0.05% Tween 20 in phosphate-buffered saline (PBST) containing 3% skim milk at 4°C overnight. Membranes were then incubated with isolated immune plasma (1:1000) at 25°C for 1 h, washed with PBST three times, and incubated with anti-chicken IgY peroxidase rabbit antibody (Sigma-Aldrich, St. Louis, MO, USA) at 25°C for 1 h and washed with PBST three times. Finally, the peroxidase signal was visualized by incubating the membrane with Immobilon Western Chemiluminescent Horseradish Peroxidase (HRP) Substrate (Merck) for 5 min at 25°C.

### 2.9 Enzyme-linked immunosorbent assay

Antibody titers of each immune plasma sample were determined via enzyme-linked immunosorbent assay (ELISA). Recombinant CP-PDs (100 ng/well) were coated on wells of 96-well plates (Sumitomo Bakelite Co. Ltd., Tokyo, Japan) and incubated in carbon-bicarbonate buffer (pH 9.8) at 4°C overnight. Thereafter, each well was blocked with PBST-containing 1% bovine serum albumin at 37°C for 2 h. After blocking, 8,000-, 16,000-, and 32,000-fold PBS-diluted immune plasma was added to each well and incubated at 25°C for 30 min. Then, wells were washed five times with PBST and incubated at 37°C with anti-chicken IgY[IgG](H+L)-HRP, Goat (Bethyl Laboratories, Inc., Montgomery, TX, USA) for 1 h. 3, 3’, 5, 5’-tetramethylbenzidine (TMB) One Component HRP Microwell Substrate (Bethyl Laboratories, Inc.) was added to each well and incubated at 37°C for 15 min. After adding 100 μL of 0.18 M H_2_SO_4_ to each well, sample absorbance was measured at 450 nm. The plasma from control chickens was diluted 2,000-fold and its absorbance was measured as previously described. The cutoff value was set at OD_450_ = 0.18, and the antibody titer was indicated as the maximum dilution rate.

### 2.10 Enzyme activity assay

Enzymatic activities of rCP-PDs were assessed using 1, 5, and 10 μg of recombinant proteins, and substrates and CP inhibitors (Cathepsin L Inhibitor) from a commercial SensoLyte Rh110 Cathepsin L Assay kit (AnaSpec, Inc., Fremont, CA, USA) in accordance with the manufacturer’s instructions. Fluorescence excitation and emission values at 490 nm and 520 nm, respectively, were measured.

### 2.11 *In vitro* feeding assay

*In vitro* feeding assays were performed as previously described [12]. Briefly, heparinized chicken blood was collected from healthy chickens maintained at the Field Science Center for the Northern Biosphere, Hokkaido University and incubated at 40°C before use. Plasma samples from rCP-PD-immunized and control chickens were pooled with those of their respective groups. Whole blood cells and plasma from heparinized blood were separated by centrifugation at 2,000 × g for 10 min. Then, the separated plasma was replaced with the pooled plasma obtained from immunized or control chickens. Unfed PRMs of mixed developmental stages were collected within *in vitro* feeding devices, and blood feeding was performed for 4 h at 40°C in dark and humid conditions with shaking at 100 rpm. Subsequently, only blood fed PRMs were collected using Pasteur pipettes, with mites maintained at 25°C in 60% humidity. The mortality of blood fed PRMs was monitored daily for one week. Anti-PRM effects of rCP-PDs were evaluated based on PRM survival rates (number of dead PRMs/number of blood-fed PRMs). For each *in vitro* feeding assay, we used the same lot of PRMs, which were collected at the same time, stored under the same conditions, and maintained in a single tube, to reduce the bias as much as possible.

### 2.12 Statistical analysis

Kaplan–Meier curves were generated and a log-rank test was performed to compare PRM mortality levels of immunized and control groups after *in vitro* feeding. Additionally, between-group comparisons of PRM mortality were performed daily using the Fisher’s exact test. Odds ratios and 95% confidence intervals (CI) were calculated. All statistical analyses were performed using EZR [31] statistical software. Statistical significance was set at *P* < 0.05 for Fisher’s exact test and *P* < 0.01 for log-rank test.

## 3 Results

### 3.1 Identification and genetic characterization of CP genes of TFMs and NFMs

ORFs of the *CP* genes from TFMs and NFMs collected and morphologically and genetically characterized from poultry farms in Myanmar [29], were determined. Nucleotide sequences of TFM and NFM ORFs completely matched, and the nucleotide and deduced amino acid sequences were highly homologous to those of PRM (Table 1). A BLAST search confirmed that deduced amino acid sequences of CPs of TFMs and NFMs were predicted to be CPs. CPs consist of a signal peptide at 1–16 positions, inhibitor domain at 246–302 positions, and peptidase C1 domain at 331–544 positions. Peptidase C1 domains, which are functional domains required for the enzymatic activity of CPs, were highly conserved among PRMs considered (89.5% similarity). Although differences in CP amino acid sequences were observed when those of TFM/NFM and PRM compared, four predicted active sites were conserved (S1 Fig). Therefore, proteins from TFM and NFM were predicted to be CPs with enzymatic activity similar to that of PRM, which functions via inhibitor domain cleavage.

**Table 1:** Comparison of cysteine protease sequences of poultry red mites (PRM), northern fowl mites (NFM), and tropical fowl mites (TFM).

		Amino acid sequence (%)		
		PRM	TFM	NFM
**Nucleotide sequence similarity (%)**	**PRM**	-	74.0	74.0
	**TFM**	73.0	-	100
	**NFM**	73.0	100	-

**Fig 1:**
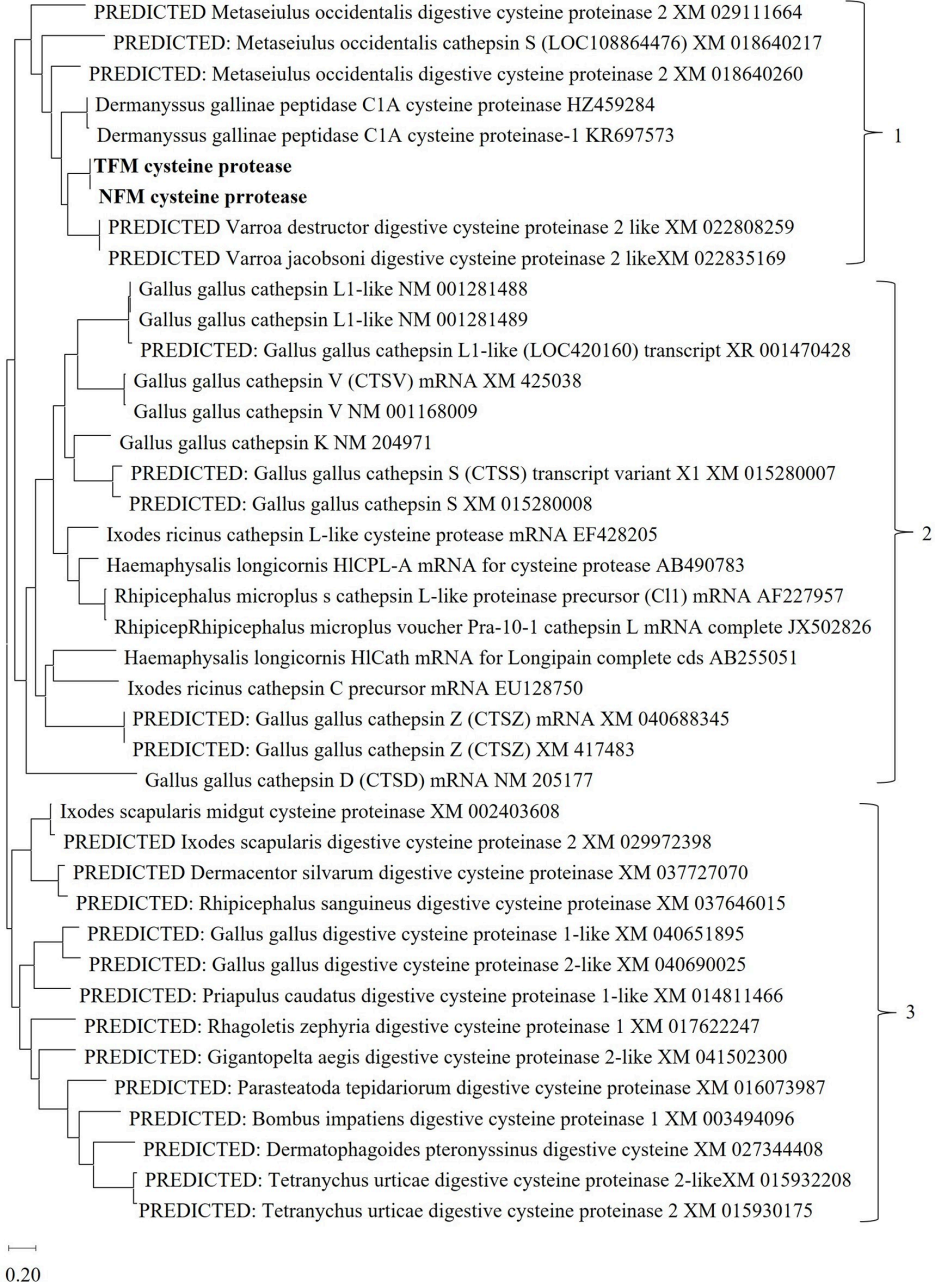
Phylogenetic analysis of cysteine proteases (CPs). The *CP* sequences of poultry red mites (PRMs), tropical fowl mites (TFMs), northern fowl mites (NFMs), other arthropods (including other mites and ticks), chickens, and similar species such as insects and invertebrates were used for analysis. The phylogenetic tree was constructed using the maximum-likelihood method with MEGA X software. The numbers on the right indicate clusters. Cluster 1 contains digestive cysteine proteases from TFM and NFM (bold) along with that of PRM and other mites. Cluster 2 comprises cysteine proteases and cathepsins D, L, K, S, V and Z of chickens and cysteine proteases of some ticks. Cluster 3 contains cysteine proteases of mites other than those of cluster 1.

To compare genetic characteristics of the *CP* genes of TFM, NFM, and PRM, phylogenetic analysis of various *CP* genes from arthropods, chickens, and other species, such as insects and invertebrates, was performed (Fig 1, S2 Table). *CP* genes from TFM and NFM belonged to cluster 1 with those from PRMs and other mite species such as *Metaseiulus occidentalis* and *Varro*a mites. In cluster 2, cathepsin L-like *CP* genes, which contribute to hemoglobin digestion in some ticks, and other *CP* (cathepsin D, K, L, S, V, and Z) genes from chickens were included. Clusters 1 and 2 formed the same rooted clade. Other digestive *CP* genes in ticks and some insects, apart from those in clusters 1 and 2, belonged to cluster 3. Thus, *CP* genes of mites, including those of TFM and NFM, seem to be related to cysteine digestion in various species.

### 3.2 Enzymatic activities of recombinant PRM, TFM, and NFM CP-PD proteins

**Fig 2:**
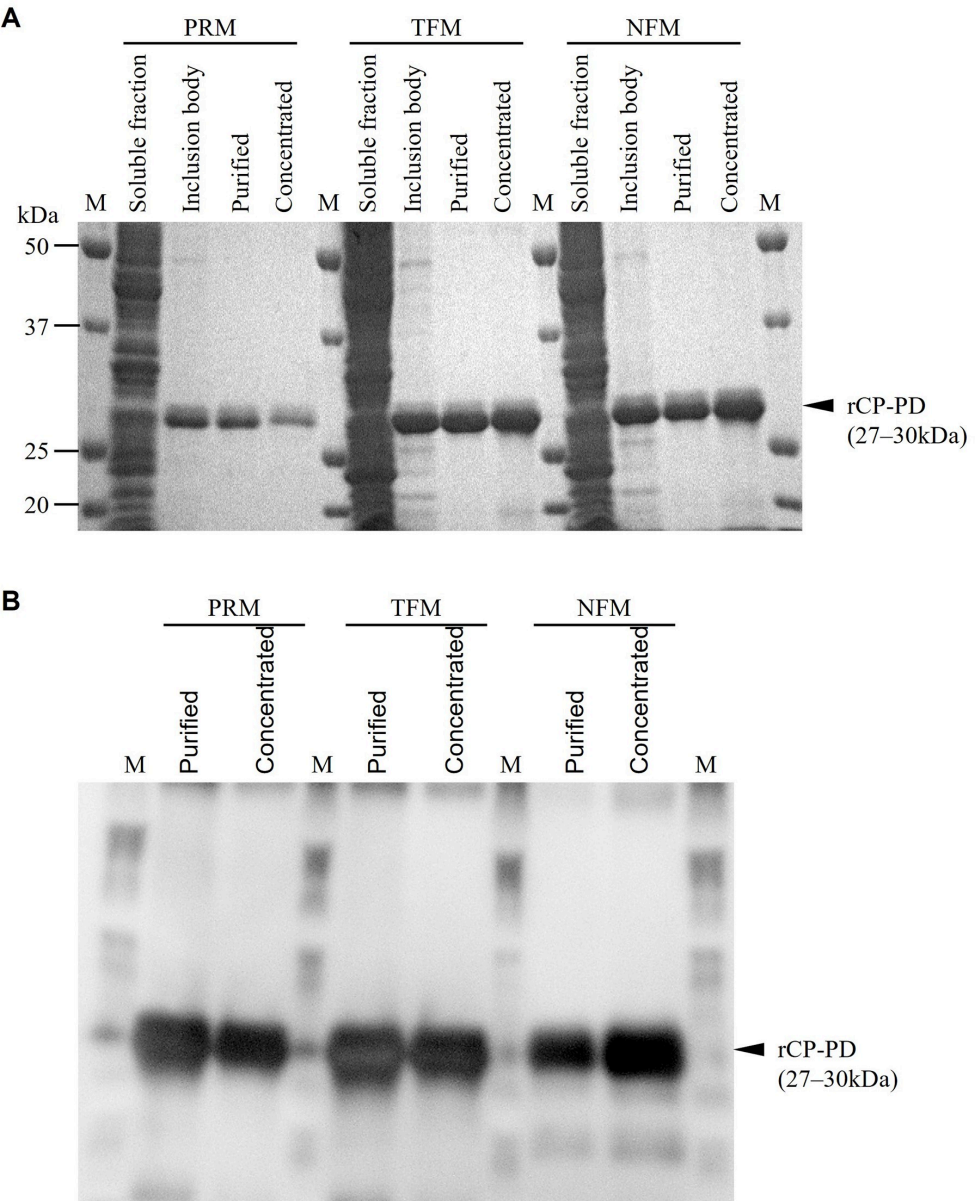
Expression and purification of recombinant cysteine protease-peptidase domain (CP-PD) proteins. The peptidase domain regions of cysteine proteases from poultry red mites (PRMs), tropical fowl mites (TFMs), northern fowl mites (NFMs) were expressed and purified as recombinant proteins fused with histidine tag, and named rCP-PD PRM, rCP-PD TFM and rCP-PD NFM, respectively. rCP-PDs were expressed using an *E*. *coli* expression system, with recombinant proteins purified from the inclusion body fraction using metal affinity resin. The expression and purity of rCP-PDs confirmed via sodium dodecyl sulfate-polyacrylamide gel electrophoresis (A) and western blotting (B) are shown. M, Marker (Precision Plus Protein™ All Blue Prestained Protein Standards, Bio-Rad, CA, USA).

**Fig 3:**
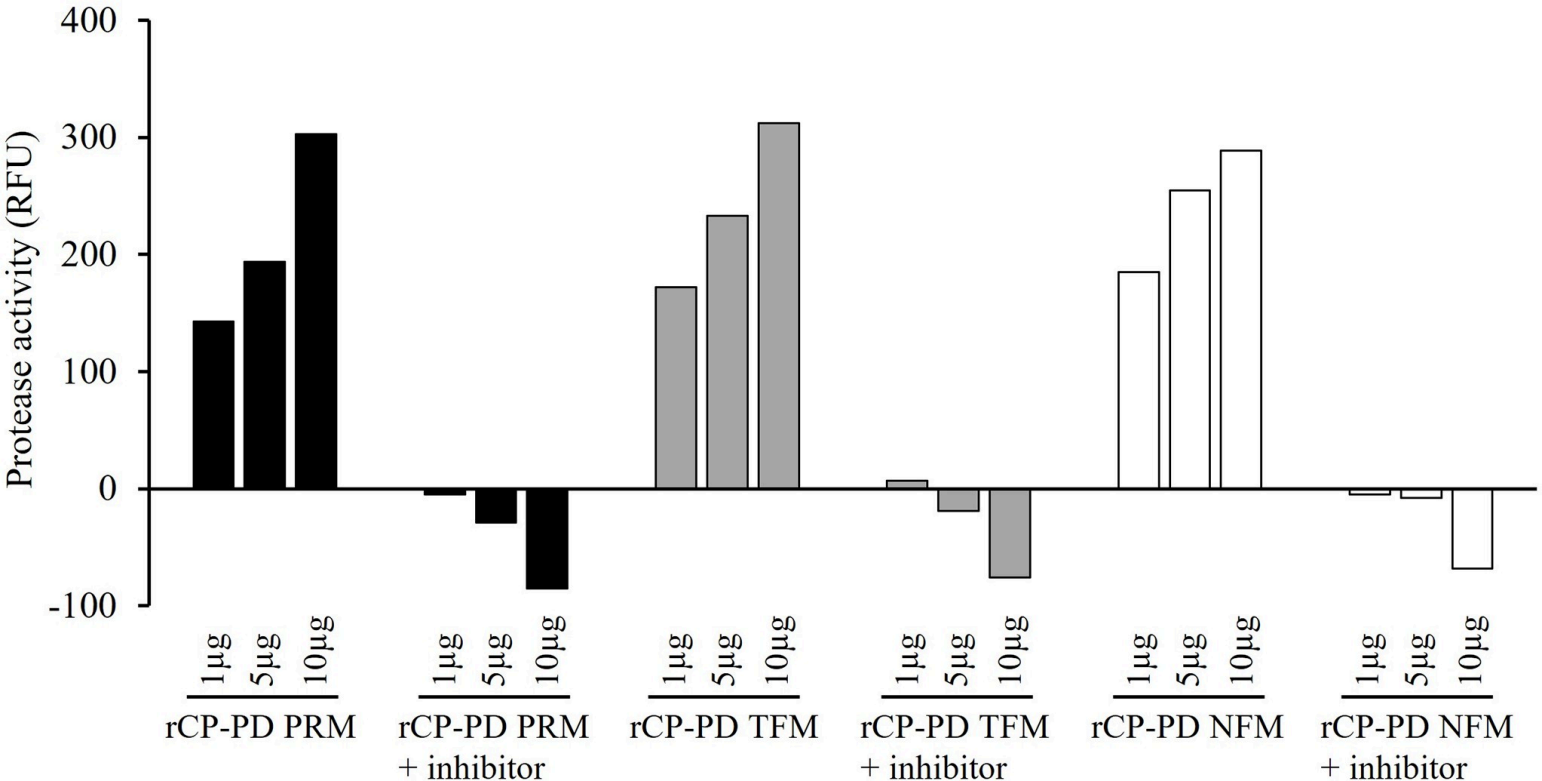
Enzyme activity of recombinant cysteine protease peptidase domains (rCP-PDs). The enzyme activity of rCP-PDs from poultry red mites (PRMs), tropical fowl mites (TFMs), northern fowl mites (NFMs) were assessed using s commercial SensoLyte Rh110 Cathepsin L Assay Kit (AnaSpec, Inc., CA, USA). In addition, enzymatic activities were assessed in the presence of a cysteine protease inhibitor. The x-axis indicates the rCP-PD quantities used in assays. Protease activity is indicated using relative fluorescence units (RFU).

PDs of CPs of PRMs, TFMs, and NFMs were prepared as the following recombinant proteins: rCP-PD PRM, rCP-PD TFM, and rCP-PD NFM, respectively. CPs were fused with a His-tag and expressed using an *E*. *coli* expression system. The sequences of CP-PDs from TFM and NFM completely matched; however, we prepared recombinant proteins using both TFM and NFM sequences to ensure the reproducibility of subsequent experiments. All rCP-PDs were purified from insoluble fractions via affinity chromatography. Protein purity was confirmed via SDS-PAGE and western blotting (Fig 2A and 2B). A previous report revealed a cathepsin -L-like enzyme activity for the rCP-PD of PRM [18]; therefore, in this study, we functionally assessed rCP-PDs using a commercial fluorescent substrate (Fig 3). We observed that all rCP-PDs had dose-dependent cathepsin L-like enzyme activity. Further, enzymatic activities were inhibited by the addition of a cathepsin L inhibitor. These results suggest that rCP-PDs have cathepsin L-like CP activities similar to that of the CP of PRM.

### 3.3 Cross-reactivities of antibodies produced via immunization with rCP-PDs

**Fig 4:**
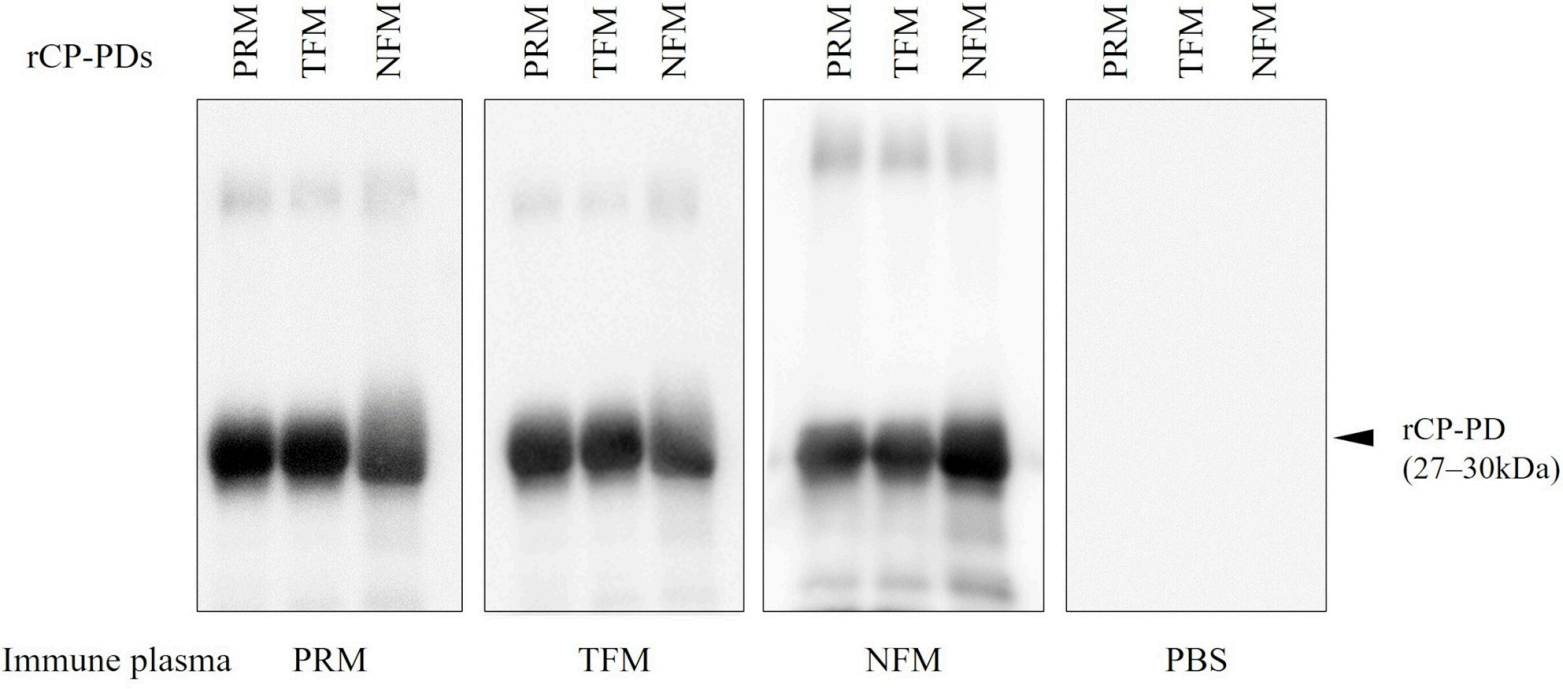
The production of specific antibodies in the plasma of chickens immunized with recombinant cysteine protease peptidase domains (rCP-PDs). Four chickens per group were immunized with rCP-PDs. Then, plasma was isolated from each immunized chicken. The presence of antibodies specific to rCP-PDs was detected by western blotting. The cross-reactivity of immune plasma of each immunized group against rCP-PDs was detected. The arrowhead indicates the predicted molecular weight of rCP-PDs (27–30 kDa).

To examine the potential usefulness of CPs as antigens against avian hematophagous mites, plasma samples were isolated from chickens immunized with each rCP-PD. Increased antibody titers in each immunized group were confirmed via ELISA (Table 2). Western blot analysis revealed that each immunized group produced specific antibodies against rCP-PDs. Moreover, plasma from chickens immunized with rCP-PD PRM, rCP-PD TFM, or rCP-PD NFM recognized rCP-PDs from different mite species (Fig 4), indicating CPs are likely useful as antigens for a universal vaccine targeting avian hematophagous mites.

**Table 2:** Antibody titers of plasma samples derived from chickens immunized using recombinant cysteine proteases.

Group	Chicken	Antibody titer
Control	C1	< 2,000
	C2	< 2,000
	C3	< 2,000
	C4	< 2,000
Immunized against the PRM rCP-PD	PRM1	> 32,000
	PRM2	> 32,000
	PRM3	> 32,000
	PRM4	16,000
Immunized against the TFM rCP-PD	TFM1	16,000
	TFM2	> 32,000
	TFM3	16,000
	TFM4	> 32,000
Immunized against the NFM rCP-PD	NFM1	> 32,000
	NFM2	16,000
	NFM3	16,000
	NFM4	> 32,000

rCP-PD, recombinant peptidase domains of cysteine protease; PRM, poultry red mite; TFM, tropical fowl mite; NFM, northern fowl mite

### 3.4 Assessment of the acaricidal activity of plasma from chickens immunized with each rCP-PD

**Fig 5:**
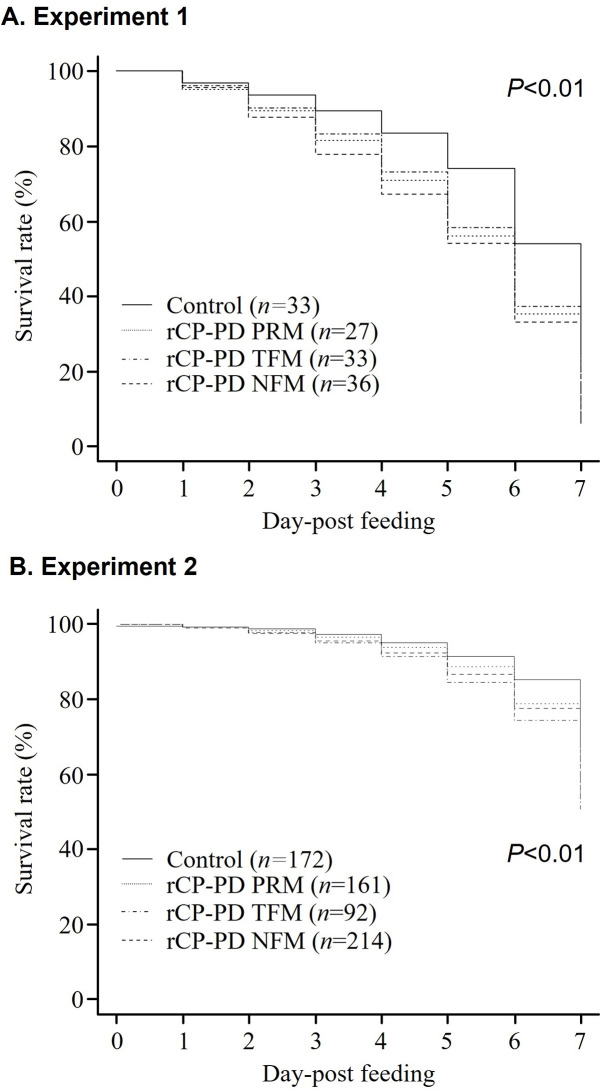
Acaricidal potential of plasma obtained from chickens immunized with recombinant cysteine protease peptidase domains (rCP-PDs). Artificial blood feeding of poultry red mites (PRMs) was performed via an *in vitro* feeding assay. The survival rate of PRMs fed with immune plasmas from each immunized and control group was accessed daily for one week. The *in vitro* feeding assays were performed 2 times. Numbers of PRMs used in this study are as follows: (A) Experiment 1: fed immune plasma, *n* = 27 (rCP-PD PRM), *n* = 33 (rCP-PD TFM), *n* = 36 (rCP-PD NFM), fed control plasma: *n* = 33; (B) Experiment 2: fed immune plasma: *n* = 161 (rCP-PD PRM), *n* = 92 (rCP-PD TFM), *n* = 214 (rCP-PD NFM), fed control plasma: *n* = 172. N The number of dead PRMs recorded and plotted to generate Kaplan–Meier survival curves are shown. Statistical analysis was performed using the log-rank test. Values of *P* < 0.01 indicate statistical significance.

Previous reports revealed that the survival rate of PRMs fed on blood containing plasma from chickens immunized with recombinant CP, which was prepared from the whole region without signal peptides, was significantly decreased [14, 18]. In this study, to assess the feasibility of CPs as antigens for a universal vaccine against avian hematophagous mites, *in vitro* feeding assays were performed to determine the mortality of PRMs fed with immune plasma of each type. TFMs are not prevalent and the distribution of NFMs is sporadic in Japan; therefore, we assessed whether CPs may be used as antigens against PRMs. In the assays, mixed stage PRMs were fed the immune plasma against each rCP-PD. The *in vitro* feeding assay was performed twice. To evaluate the acaricidal potential of immunization with rCP-PDs, mortality rates of mites fed on the sera of each immunized group were compared with those of the control group using Fisher’s exact and Kaplan–Meier log rank tests. In the first feeding experiment, mortality rates of PRMs fed plasma from each rCP-PD-immunized group increased throughout the monitoring period (Table 3). Survival of experimental versus control mites significantly differed, as follows: on day 5 after feeding on rCP-PD PRM-immunized plasma, on days 5 and 7 after feeding on rCP-PD TFM-immunized plasma, and on days 2–5 and 7 after feeding on rCP-PD NFM-immunized plasma. A comparison of Kaplan–Meier curves revealed that survival rates of PRMs fed plasma of chickens immunized with each rCP-PD were significantly lower than those of PRMs fed control group-derived plasma (Fig 5A). Similarly, in the second experiment, we observed that survival rates of PRMs fed plasma of immunized chickens was significantly lower than that of the control group (Fig 5B). Fisher exact and odds ratio analysis showed that mortality rates of PRMs were significantly increased after feeding on immune plasma. This increase was observed on days 6 and 7 in the rCP-PD PRM group, days 2 and 5–7 in the rCP-PD TFM group, and days 1, 2, and 7 in the rCP-PD TFM group (Table 4). Taken together, these data suggest that immunization against all rCP-PDs produces acaricidal activity in PRMs, suggesting that CPs are likely suitable antigens for universal vaccine development targeting a broad range of avian hematophagous mites.

**Table 3:** Mortality of PRMs fed plasma of chickens immunized with recombinant cysteine proteases from different species of mites (Experiment 1).

	Days post-feeding						
	1	2	3	4	5	6	7
**Control group (*n* = 33)**							
No. of dead PRMs post-feeding	7	7	7	9	11	18	18
Mortality (%)	21.2	21.2	21.2	27.3	33.3	54.5	54.5
**Immunized group (rCP-PD PRM) (*n* = 27)**							
No. of dead PRMs post-feeding	9	10	12	14	17	20	21
Mortality (%)	26.1	30.4	39.1	47.8	56.5	69.6	73.9
Chi-square	0.581	1.135	2.708	2.826	4.115	1.670	2.575
*P* value	0.382	0.251	0.093	0.065	0.036*	0.178	0.102
Odds ratio	1.83	2.15	2.91	2.81	3.32	2.34	2.86
95% confidence interval	0.50–7.00	0.60–8.12	0.84–10.85	0.86–9.69	1.03–11.31	0.70–8.46	0.83–11.02
**Immunized group (rCP-PD TFM) (*n* = 33)**							
No. of dead PRMs post-feeding	9	12	13	16	20	24	28
Mortality (%)	17.9	28.6	32.1	42.9	57.1	67.9	82.1
Chi-square	0.082	1.183	1.794	2.318	3.893	1.636	5.811
*P* value	0.775	0.277	0.18	0.127	0.048*	0.2	0.015*
Odds ratio	1.385	2.098	2.381	2.474	3.022	2.194	4.554
95% confidence interval	0.39–5.14	0.63–7.51	0.72–8.47	0.81–8.02	1.01–9.51	0.71–7.12	1.29–18.92
**Immunized group (rCP-PD NFM) (*n* = 36)**							
No. of dead PRMs post-feeding	11	18	20	20	21	28	31
Mortality (%)	29.0	45.2	51.6	51.6	54.8	77.4	83.9
Chi-square	0.37	4.992	7.145	4.551	3.38	3.202	6.87
*P* value	0.422	0.023*	0.006*	0.028*	0.053*	0.072	0.007*
Odds ratio	1.622	3.641	4.533	3.273	2.757	2.87	5.038
95% confidence interval	0.48–5.79	1.16–12.61	1.44–15.77	1.09–10.44	0.95–8.41	0.92–9.57	1.44–20.82

*Statistically significant based of a significance level set at *P* < 0.05

rCP-PD, recombinant peptidase domains of cysteine protease; PRM, poultry red mite; TFM, tropical fowl mite; NFM, northern fowl mite

**Table 4:** Mortality of PRMs fed plasma of chickens immunized with recombinant cysteine proteases from different species of mites (Experiment 2).

	Days post-feeding						
	1	2	3	4	5	6	7
**Control group (*n* = 172)**							
No. of dead PRMs post-feeding	2	6	11	17	20	23	34
Mortality (%)	1.2	3.5	6.4	9.9	11.6	13.4	19.8
**Immunized group (rCP-PD PRM, *n* = 161)**							
No. of dead PRMs post-feeding	7	11	14	18	26	36	50
Mortality (%)	4.4	6.8	8.7	11.3	16.1	22.4	31.1
Chi-square	2.11	1.29	0.34	0.04	1.07	4.01	5.03
*P* value	0.095	0.214	0.533	0.724	0.267	0.043*	0.022*
Odds ratio	3.84	2.02	1.39	1.14	1.46	1.86	1.82
95% confidence interval	0.71–38.53	0.66–6.83	0.56–3.50	0.53–2.47	0.74–2.89	1.01–3.47	1.07–3.12
**Immunized group (rCP-PD TFM, *n* = 92)**							
No. of dead PRMs post-feeding	4	9	12	15	21	22	30
Mortality (%)	4.3	9.8	13.0	16.3	22.8	23.9	32.6
Chi-square	1.49	3.33	2.54	1.75	4.90	3.99	4.70
*P* value	0.187	0.049*	0.107	0.165	0.020*	0.038*	0.024*
Odds ratio	3.84	2.98	2.18	1.77	2.24	2.03	1.95
95% confidence interval	0.53–43.25	0.91–10.56	0.84–5.74	0.77–4.00	1.07–4.66	1.00–4.10	1.05–3.62
**Immunized group (rCP-PD NFM, *n* = 214)**							
No. of dead PRMs post-feeding	12	20	23	29	39	45	68
Mortality (%)	5.6	9.3	10.7	13.6	18.2	21.0	31.8
Chi-square	4.19	2.63	1.73	0.89	2.71	3.34	6.46
*P* value	0.026*	0.024*	0.151	0.343	0.087	0.059	0.010*
Odds ratio	5.03	0.84	1.75	1.42	1.69	1.72	1.88
95% confidence interval	1.09–46.91	1.06–8.86	0.79–4.12	0.72–2.88	0.91–3.20	0.96–3.13	1.15–3.13

*Statistically significant based of a significance level set at *P* < 0.05

rCP-PD, recombinant peptidase domains of cysteine protease; PRM, poultry red mite; TFM, tropical fowl mite; NFM, northern fowl mite

## 4 Discussion

Current control strategies targeting avian mites in poultry farms mainly rely on acaricides; however, problems such as acaricide-resistant mite selected and diminished acaricide efficacy persist. Recently, much attention has been paid to vaccination against PRMs as a promising alternative strategy for PRM control. However, other hematophagous mites such as TFMs and NFMs are prevalent in poultry farms; therefore, the development of anti-PRM vaccines effective against similar mites is encouraged to reduce economic losses to the poultry industry. The digestive enzyme Cathepsin L protease, involved in hemoglobin digestion in ticks, has been reported as a suitable candidate for developing anti-tick vaccines [28]. In PRM, a CP, Deg-CPR-1, is expressed in the midgut of PRMs, and its use as a vaccine antigen decreases the survival rate of PRMs fed plasma from immunized chickens [18]. As blood feeding is a common behavior essential for the growth of these mites, Deg-CPR-1 could be a useful antigen candidate for a universal vaccine against avian hematophagous mites. In the present study, we identified *CP* genes in TFMs and NFMs with nucleotide sequences closely related to those of PRMs. In addition, rCP-PDs from both TFMs and NFMs had enzymatic activities similar to that of PRMs, and immune plasma cross-reacted with the rCP-PDs from different mite species. Moreover, increased mortality was observed in PRMs fed immune plasma, even in PRMs fed plasma from chickens immunized with TFM or NFM rCP-PDs. These results suggest that CPs are potentially useful as vaccine antigens in the development of universal vaccines across mite species.

Genetic analysis revealed that the *CP* genes from TFM and NFM belong to a cluster of genes encoding digestive CPs of mite species including the CP of PRM and are clearly distinct from CP genes of other species. The cathepsin L subfamily (cathepsins L, V, K, S, F, and H), which includes papain-like CPs, contains a CP peptidase inhibitor-I29 domain (prodomain) at its N-terminus and peptidase C1A superfamily domain (mature) at its C-terminus [26]. These domains were also predicted to be present in PRM, TFM, and NFM CPs. Further, mature PDs of PRM and TFM/NFM were highly similar (89.5% similarity). The transcriptome analysis of PRM sequences revealed that reads of *CP* genes accounted for ~62% of all protease-encoding reads. Among these, 80% were cathepsin L-like proteases of the midgut [32]. In addition, expression of *PRM-CP* (*Deg-CPR-1*) has previously been observed in the midgut using laser capture microdissection and RT-PCR [18]. Collectively, these data suggested that the CP of PRM (Deg-CPR-1) is a digestive enzyme involved in blood digestion. In the present study, we were not able to analyze the expression of *CP* genes in different tissues such as the midgut of TFM and NFM due to the limited number samples available. However, rCP-PDs of TFM and NFM had enzymatic activities similar to that of PRM, and plasma from chickens immunized with TFM and NFM rCP-PDs produced acaricidal effects against PRMs, similar to that which was observed using immune plasma against rCP-PD PRM. Therefore, the CPs identified in TFMs and NFMs in this study may have functions similar to those of PRMs, although further study of expression patterns and functions of CPs in TFMs and NFMs is needed.

The potential usefulness of wide range antigens in the development of universal vaccines with broad spectrum activity against different tick species has been reported; immunization of glutathione S transferase from *Haemaphysalis longicornis* results in a 57% and 67% reduction of *Rhipicephalus microplus* and *Rhipicephalus appendiculatus* infestations of their host, respectively [33, 34]. Further, feeding of antibodies against recombinant ferritin 2 from *Ixodes persulcatus* decreased the engorgement weight of *R*. *microplus* females [35]. These molecules could be effective as vaccine antigens when a host is infested with two or more tick species. Further, they are potentially applicable in a variety of areas, a characteristic that is particularly useful tick species distributions vary. Notably, the development of an anti-tick vaccine with broad-spectrum efficacy may simplify both the commercial production of vaccines and clinical veterinary practice. Likewise, in the poultry industry, distributions of the three major species of avian mites (PRM, TFM, and NFM) varies geographically [3], with mites occasionally coexisting in the same poultry house as a mixed population. Thus, the development of a universal vaccine could be a cost-effective and simple strategy for reducing economic losses caused by avian hematophagous mites in various areas.

In the present study, immune plasma against rCP-PDs of each of three mite species showed cross-reactivity with rCP-PDs of the other two species of mites, suggesting that CPs of avian hematophagous mites are likely suitable antigens for developing a vaccine with broad-protective efficacy across mite species. To elucidate their efficacy as vaccine antigens, we assessed the acaricidal potential of rCP-PDs via *in vitro* feeding assays of PRMs. A drastic reduction in the survival rate of PRMs fed immune plasma was observed, even when plasma from chickens immunized with rCP-PDs of different mites was used. Cathepsin L proteases have been reported to play a role in hemoglobin digestion in blood-feeding arthropods including some tick species such as *R*. *microplus*, *H*. *longicornis*, and *I*. *ricinus* [27]; therefore, they are antigen candidates for the development of anti-tick vaccines [28]. Cathepsin L proteases are potentially applicable as antigens for the development of universal vaccines against ticks. However, the present study has some limitations regarding TFM and NFM availability in Japan. As such, *in vitro* feeding assays assessed PRM but not TFM or NFM survival. To clearly highlight the potential of CPs for use as broad-spectrum antigens across avian hematophagous mites, evaluation of vaccine antigens in TFMs and NFMs will be needed.

In conclusion, this is the first report to assess the utility of CPs from avian hematophagous mites as antigen candidates for universal vaccine development. CPs from TFMs and NFMs were determined to be similar to PRM CPs and possess CP-like enzymatic activity. In addition, immune plasma against rCP-PDs from PRM, TFM, and NFM cross-reacted with rCP-PDs of different mites. Moreover, the acaricidal effects of TFM and NFM against PRMs were confirmed. To develop an effective universal vaccine for controlling avian hematophagous mites, challenge trials assessing chickens immunized with each mite are required. Prospectively, this finding will help reduce economic losses and improve animal welfare in the poultry industry.

## Supporting information

S1 Fig Deduced amino acid sequences of cysteine proteases (CPs) from TFMs and NFMs aligned with that of PRMs. The structure of the amino acid sequence of CPs is as follows: signal peptide (dashed white box) at positions of 1–16 in TFMs and NFMs and 1–18 in PRMs, peptidase inhibitor domain (grey box) at 246–302 in TFM and NFM and 249–305 in PRM, and peptidase domain (PD, white box) at 331–544 in TFM and NFM and 335–547 in PRM. The black arrowhead indicates predicted active sites for peptidase activities. Poultry red mites (PRMs), tropical fowl mites (TFMs), northern fowl mites (NFMs).
(TIF)

S2 Fig Uncropped versions of images included in Fig 2A. (TIF)

S3 Fig Uncropped versions of images included in Fig 2B. (TIF)

S4 Fig Uncropped versions of images included in Fig 4. (TIF)

S1 Table List of primers used for the amplification of cysteine protease genes. (DOCX)

S2 Table A list of *cysteine protease* genes used for the phylogenetic analysis shown in Fig 1. (XLSX)
