# Supplementary figures and images for: Characterization of cysteine proteases from poultry red mite, tropical fowl mite, and northern fowl mite to assess the feasibility of developing a broadly efficacious vaccine against multiple mite species
Source: PLoS One. 2023 Jul 13;18(7):e0288565. doi: 10.1371/journal.pone.0288565 (PMC10343161; doi:10.1371/journal.pone.0288565)

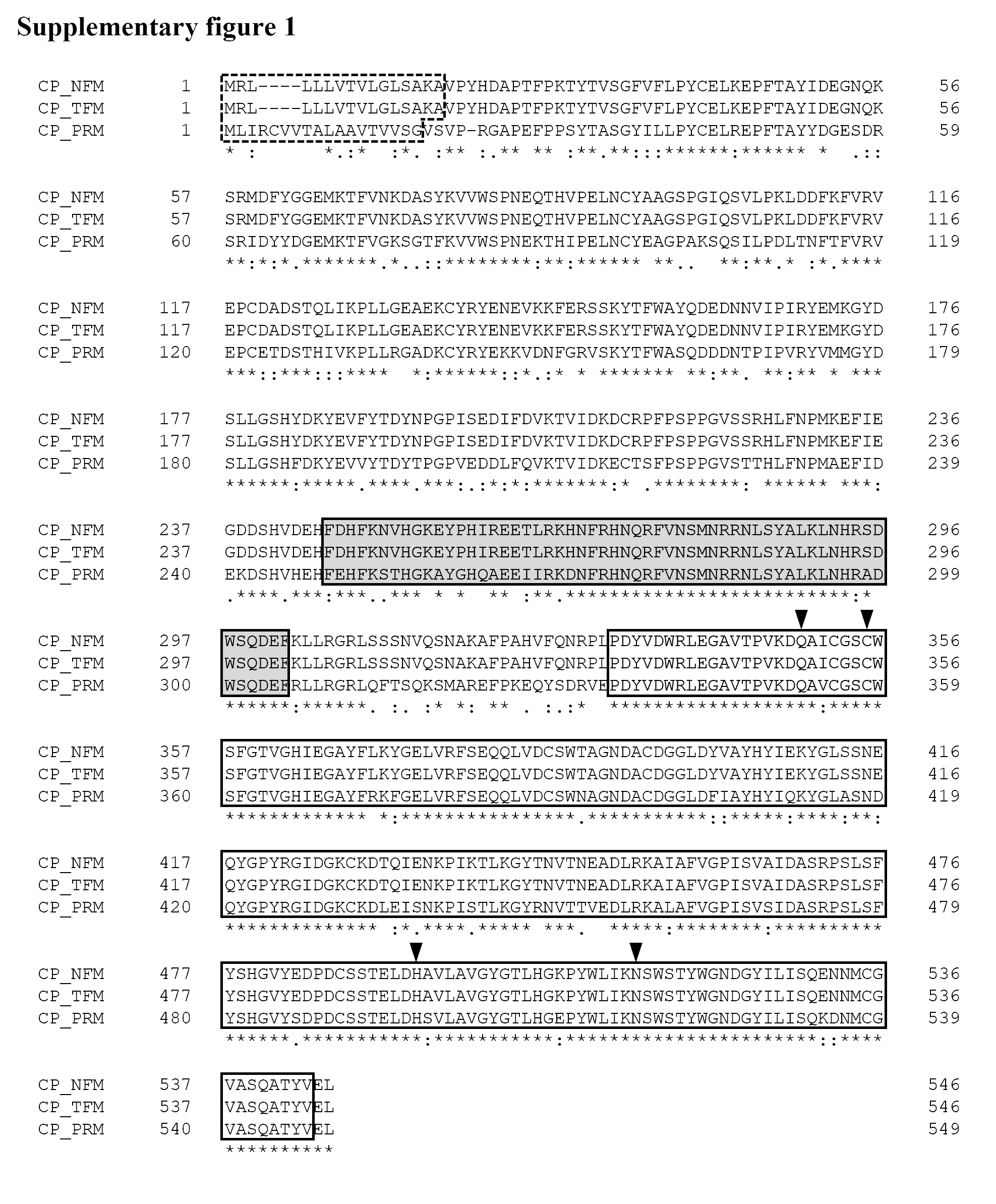

Supplement: S1 Fig — The structure of the amino acid sequence of CPs is as follows: signal peptide (dashed white box) at positions of 1–16 in TFMs and NFMs and 1–18 in PRMs, peptidase inhibitor domain (grey box) at 246–302 in TFM and NFM and 249–305 in PRM, and peptidase domain (PD, white box) at 331–544 in TFM and NFM and 335–547 in PRM. The black arrowhead indicates predicted active sites for peptidase activities. Poultry red mites (PRMs), tropical fowl mites (TFMs), northern fowl mites (NFMs). (TIF) [file pone.0288565.s001.tif]

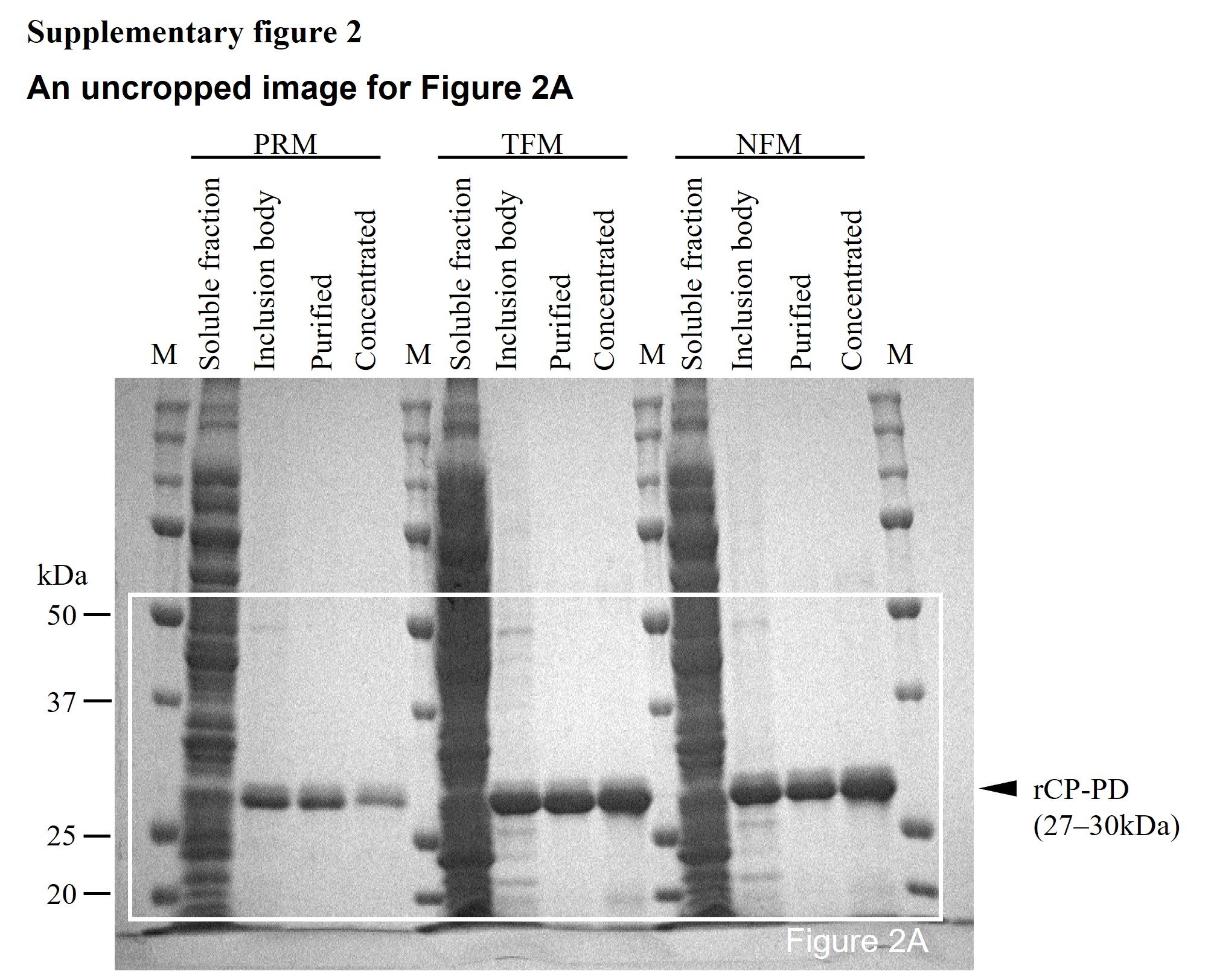

Supplement: S2 Fig — (TIF) [file pone.0288565.s002.tif]

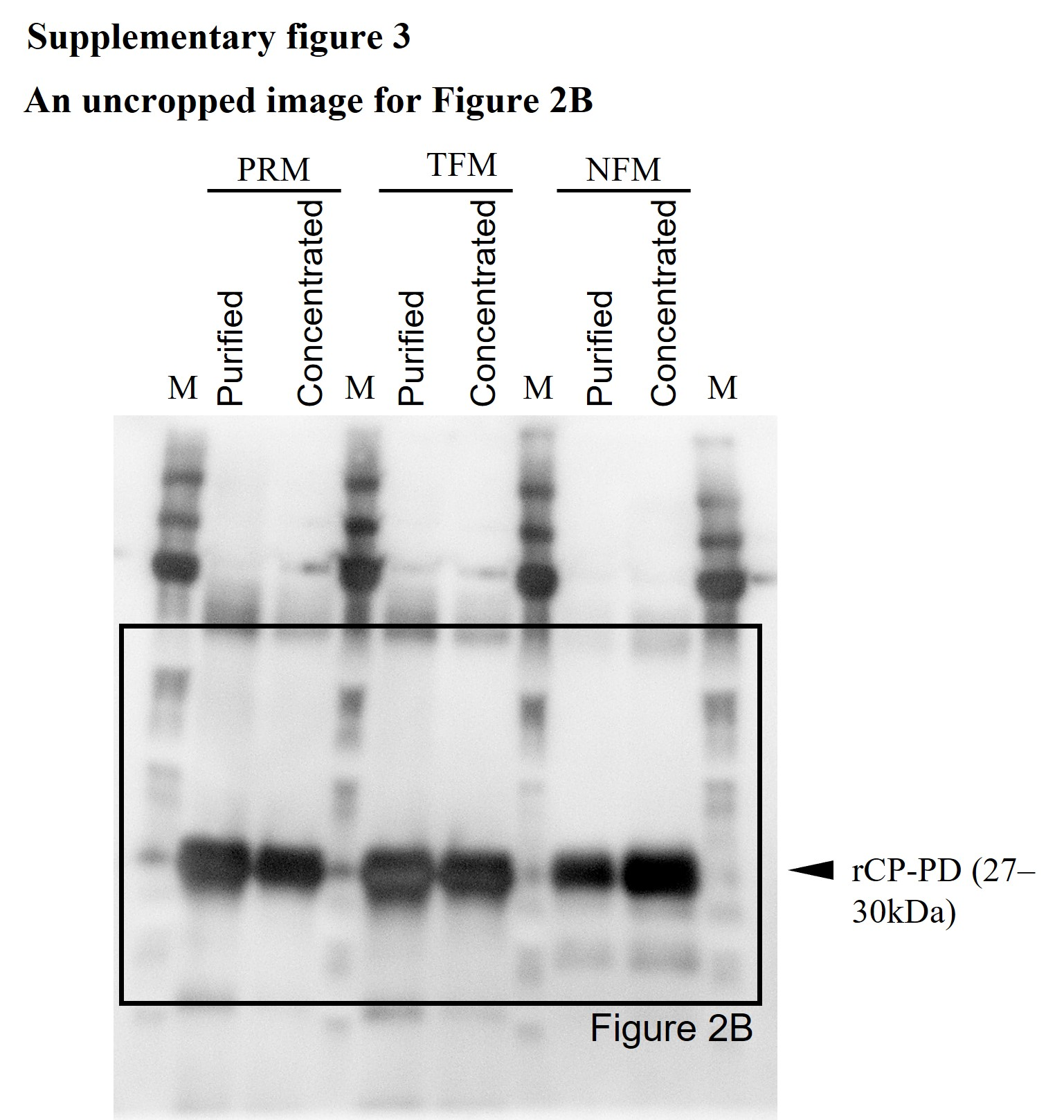

Supplement: S3 Fig — (TIF) [file pone.0288565.s003.tif]

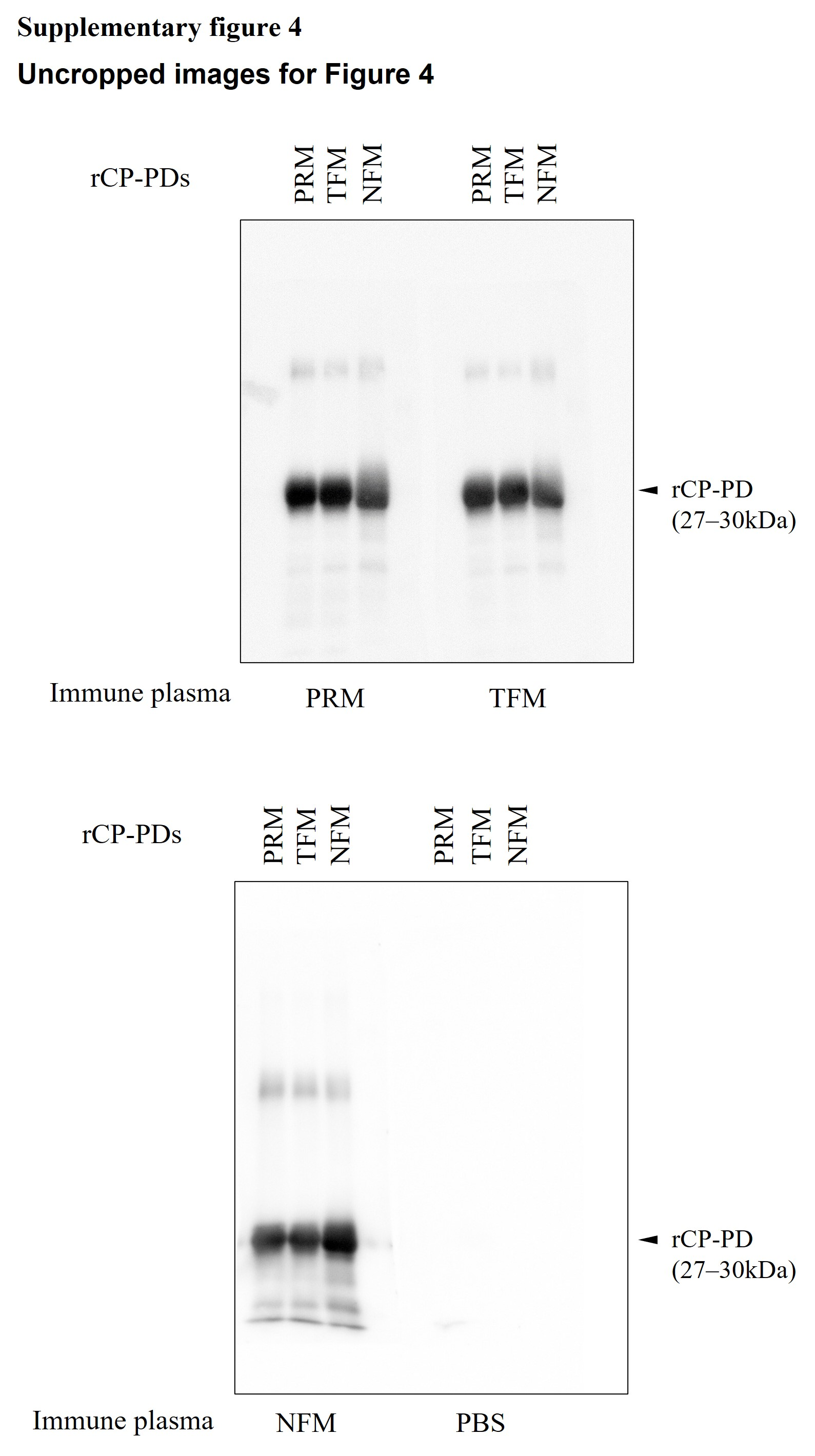

Supplement: S4 Fig — (TIF) [file pone.0288565.s004.tif]
