# Supplementary material for: Characterization of cysteine proteases from poultry red mite, tropical fowl mite, and northern fowl mite to assess the feasibility of developing a broadly efficacious vaccine against multiple mite species
Source: PLoS One. 2023 Jul 13;18(7):e0288565. doi: 10.1371/journal.pone.0288565 (PMC10343161; doi:10.1371/journal.pone.0288565)
Supplement: S1 Table — (DOCX) [file pone.0288565.s005.docx]

| Supplementary table 1. List of primers used for the amplification of cysteine protease genes in this study | | | |
| --- | --- | --- | --- |
| Primer | Mite species | Intended use | Sequences (5´-3´) |
| CP outer -F | NFM & TFM | Partial gene amplification | TACRAHGGMGAGATGAARACMTTC |
| CP outer- R |  |  | TCKAGTKMCGTCGARCTGCA |
| CP inner -F |  |  | CTCYAARTAYACSTTCTGGGC |
| CP inner-R |  |  | TCCTTGCAYTTRCCRTCAATGCC |
| GSP1 | NFM & TFM | 3′RACE | GAGGGAGATGATTCGCATGT |
| GSP2 |  |  | GCAACCTTTCGTACGCTCTC |
| GSP1 | NFM & TFM | 5′RACE | TTTCACATCGAAAATA |
| GSP2 |  |  | GAGTCGGCATCACAGGGTTC |
| GSP3 |  |  | AAATCCATTCGCGATTTCTG |
| rCP-PD PRM-F | PRM | Construction for expression plasmids | AAGCATATGCCGGACTACGTCGACTGGCG |
| rCP-PD PRM-R |  |  | ATCCTCGAGCTACAGCTCGACGTAGGTTGCCTG |
| rCP-PD TFM-F | NFM & TFM |  | AAGCATATGCTTCCGGACTACGTAGACTGGCG |
| rCP-PD TFM-R |  |  | ATCCTCGAGTTAGAGTTCAACATAGGTCGCCTGTGAAG |
